# Investigate the genetic mechanisms of diabetic kidney disease complicated with inflammatory bowel disease through data mining and bioinformatic analysis

**DOI:** 10.3389/fendo.2022.1081747

**Published:** 2023-01-16

**Authors:** Xiaoyu Zhang, Huijie Xiao, Shaojie Fu, Jinyu Yu, Yanli Cheng, Yang Jiang

**Affiliations:** ^1^ Department of Gastrointestinal and Colorectal Surgery, China-Japan Union Hospital of Jilin University, Changchun, China; ^2^ Department of Nephrology, The First Hospital of Jilin University, Changchun, China; ^3^ Department of Urology, The First Hospital of Jilin University, Changchun, China

**Keywords:** diabetic kidney disease, inflammatory bowel disease, data mining, bioinformatic analysis, signaling pathway

## Abstract

**Background:**

Patients with diabetic kidney disease (DKD) often have gastrointestinal dysfunction such as inflammatory bowel disease (IBD). This study aims to investigate the genetic mechanism leading to IBD in DKD patients through data mining and bioinformatics analysis.

**Methods:**

The disease-related genes of DKD and IBD were searched from the five databases of OMIM, GeneCards, PharmGkb, TTD, and DrugBank, and the intersection part of the two diseases were taken to obtain the risk genes of DKD complicated with IBD. A protein–protein interaction (PPI) network analysis was performed on risk genes, and three topological parameters of degree, betweenness, and closeness of nodes in the network were used to identify key risk genes. Finally, Gene Ontology (GO) analysis and Kyoto Encyclopedia of Genes and Genomes (KEGG) analysis were performed on the risk genes to explore the related mechanism of DKD merging IBD.

**Results:**

This study identified 495 risk genes for DKD complicated with IBD. After constructing a protein–protein interaction network and screening for three times, six key risk genes were obtained, including matrix metalloproteinase 2 (MMP2), hepatocyte growth factor (HGF), fibroblast growth factor 2 (FGF2), interleukin (IL)-18, IL-13, and C–C motif chemokine ligand 5 (CCL5). Based on GO enrichment analysis, we found that DKD genes complicated with IBD were associated with 3,646 biological processes such as inflammatory response regulation, 121 cellular components such as cytoplasmic vesicles, and 276 molecular functions such as G-protein-coupled receptor binding. Based on KEGG enrichment analysis, we found that the risk genes of DKD combined with IBD were associated with 181 pathways, such as the PI3K-Akt signaling pathway, advanced glycation end product–receptor for AGE (AGE-RAGE) signaling pathway and hypoxia-inducible factor (HIF)-1 signaling pathway.

**Conclusion:**

There is a genetic mechanism for the complication of IBD in patients with CKD. Oxidative stress, chronic inflammatory response, and immune dysfunction were possible mechanisms for DKD complicated with IBD.

## Introduction

Diabetic kidney disease (DKD) is the most common cause of chronic kidney disease and end-stage renal disease worldwide, affecting approximately 30%–40% of people with diabetes (1). In recent years, the incidence of diabetes worldwide has increased in parallel with the prevalence of DKD due to changes in obesity rates, metabolic syndrome, and lifestyle habits (2). At the same time, due to the high medical cost and poor prognosis in the advanced stage of the disease, DKD has brought a heavy burden to the whole society and families (3).

Inflammatory bowel disease (IBD) is a chronic inflammatory disease of the gastrointestinal tract of unknown etiology. IBD has traditionally been viewed as a disease of the Western world. However, data over the past decade have shown that IBD has become a global disease with a dramatic increase in incidence and prevalence in the East (4). Due to the early onset of IBD, complex clinical symptoms (fatigue, diarrhea, and pain), and fluctuating disease course (prolonged disease course, recurrent attacks, and difficult to cure), it has a great impact on the quality of life and psychological state of patients (5). Although the precise cause and pathogenesis of IBD remain unknown and an effective cure is lacking, genetic research is now providing insight into the biological mechanisms behind the disease, which may hold promise for future therapies (6). Recently, extraintestinal symptoms and associated diseases of IBD have attracted increasing attention, most of which are related to autoimmune diseases such as type 1 diabetes mellitus, thyroid disease, and dermatitis herpetiformis (7). DKD and IBD are common and complex chronic diseases that are clinically heterogeneous and progressive. Some studies have shown that DKD and IBD are related disorders that probably share susceptibility genes. Patients with DKD are often associated with gastrointestinal dysfunction and may be more susceptible to IBD (8–10). However, the mechanism of DKD concurrent IBD remains unclear. As DKD and IBD are currently incurable, they represent a major public health challenge worldwide. Therefore, a clear knowledge of the key risk genes implicated in DKD complicated with IBD could help to identify potential therapeutic targets for the development of new drugs, in addition to the possible discovery of new potential diagnostic biomarkers for the early diagnosis of DKD complicated with IBD, while an understanding of the possible molecular mechanisms implicated in DKD and IBD could help improve the prognostic and therapeutic tools leading to a better quality of life of the patients, delaying the progression of the disease.

In recent years, with the continuous development of molecular biotechnology, bioinformatics has played an increasingly important role in exploring the molecular mechanisms of human diseases through systematic analysis of available biomedical data (11). In this study, we systematically explored the key targets and important pathways of DKD combined with IBD using bioinformatics methods, which enhanced our understanding of the underlying molecular mechanisms of DKD and IBD and provided potential targets for further research.

## Methods

### Collection of gene targets in DKD and IBD

The human genes associated with DKD and IBD were gathered from Online Mendelian Inheritance in Man (OMIM, https://omim.org/) (12), GeneCards (https://www.genecards.org/), Pharmacogenomics Knowledge Base (PharmGkb, https://www.pharmgkb.org/), Therapeutic Target Database (TTD, http://db.idrblab.net/ttd/), and DrugBank (https://www.drugbank.ca/). GeneCards is a comprehensive database that provides information on all predicted and annotated human genes, and genes with a correlation coefficient >10 are filtered as relevant genes (13). PharmGKB is a comprehensive resource on how human genetic variation leads to variation in drug response (14). TTD is a database that provides information on known and explored therapeutic proteins and targeted diseases (15). DrugBank, a comprehensive bioinformatics database, also provides relevant information on disease targets (16). The search terms “diabetic kidney disease” and “inflammatory bowel disease” were used to retrieve data from the above five databases, respectively.

### Identification of the risk genes for DKD with IBD

We gathered the DKD- and IBD-related genes. The potential risk genes for DKD and IBD were identified from the above-shared genes.

### Protein–protein interaction network

The risk genes of DKD and IBD were imported into the STRING database to obtain their interaction relationship. STRING (https://string-db.org/, version 11.0) is a database containing known and predicted PPI, which gathers information using bioinformatics strategies (17). These species are restricted to “*Homo sapiens*,” and a PPI with a confidence score >0.9 was chosen for this study.

### Network construction

The PPI network of risk genes for DKD with IBD was built by linking them to their interacting genes. Next, the network visualization software Cytoscape version 3.4.2 (http://www.cytoscape.org/) was used to present the network. Lastly, Network Analyzer was used to calculate four topological parameters of each node in the network, including the degree, betweenness centrality, closeness centrality, and clustering coefficient (18). Nodes with values above the median for all four topological parameters were selected to construct a subnetwork, where another selection was finally performed to obtain the core risk genes for DKD and IBD.

### GO and KEGG pathway enrichment analysis

To further understand the role of DKD and IBD risk genes in biological process (BP), cellular component (CC), and molecular function (MF), we used the Gene Ontology (GO) database (http://geneontology.org/) to clarify the possible biological mechanisms. KEGG (https://www.kegg.jp/) is a database for extracting biological information on functional classification, annotation, and enriched pathways of various genes. In this study, we used an R-package-Bioconductor clusterProfiler for GO and KEGG enrichment analysis. The R-package-Bioconductor clusterProfiler is widely used to automate biological term classification and enrichment analysis of gene clusters (19).

## Results

**Figure 1:**
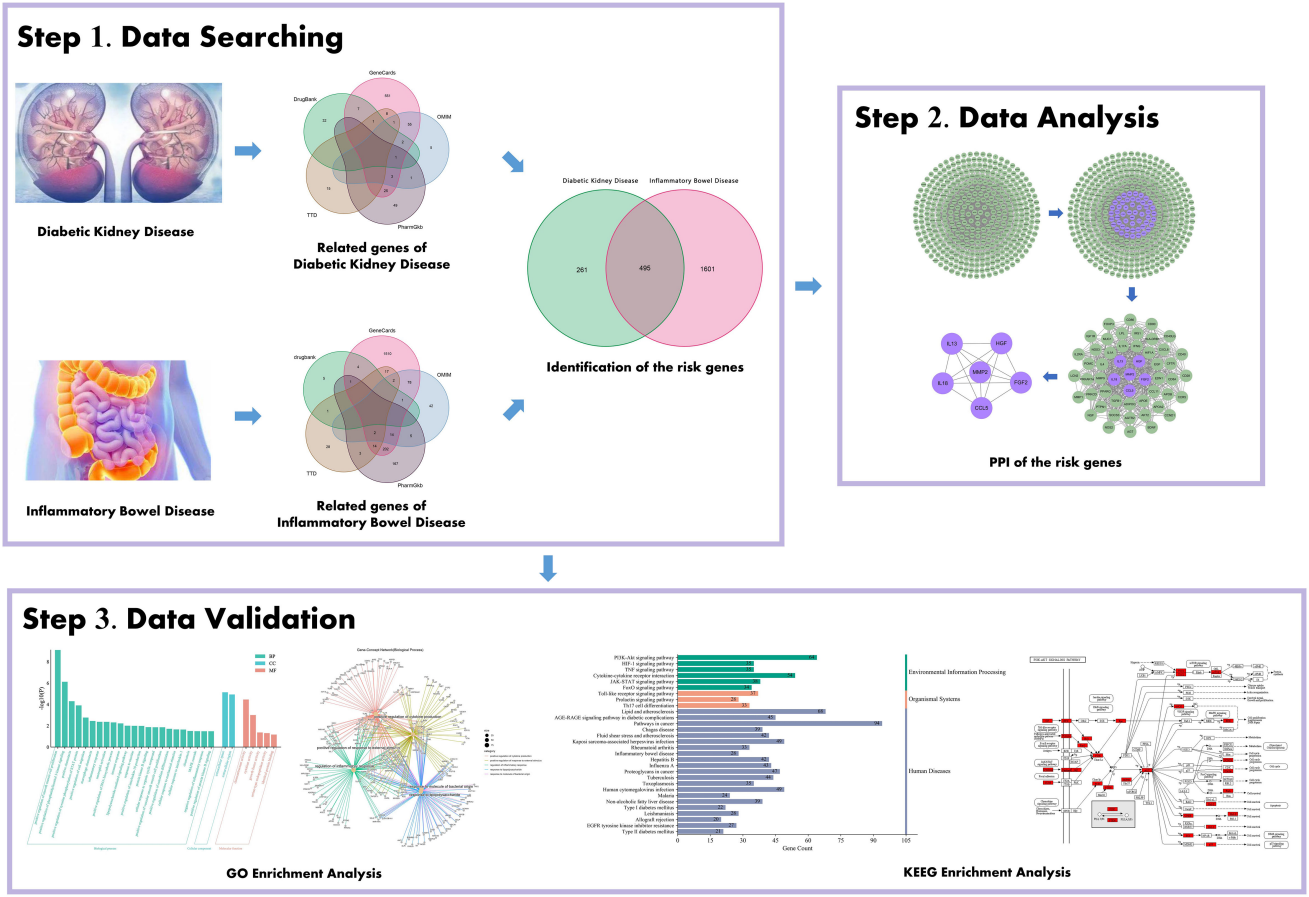
Schematic illustration showing the investigative process undertaken in the present study. This investigation is organized into three sections: Step 1, collection of gene targets/identification of the risk genes for diabetic kidney disease (DKD) with inflammatory bowel disease (IBD); Step 2, mining the risk genes for DKD with IBD’s PPI network; Step 3, probing the pathways and biological process, gene-disease enrichment analysis related to obtained important intersection protein/genes, and then validation with a literature review.

The flowchart of the study based on data mining and bioinformatic analysis is presented in **Figure 1**.

### Collection of gene targets in DKD and IBD

**Figure 2:**
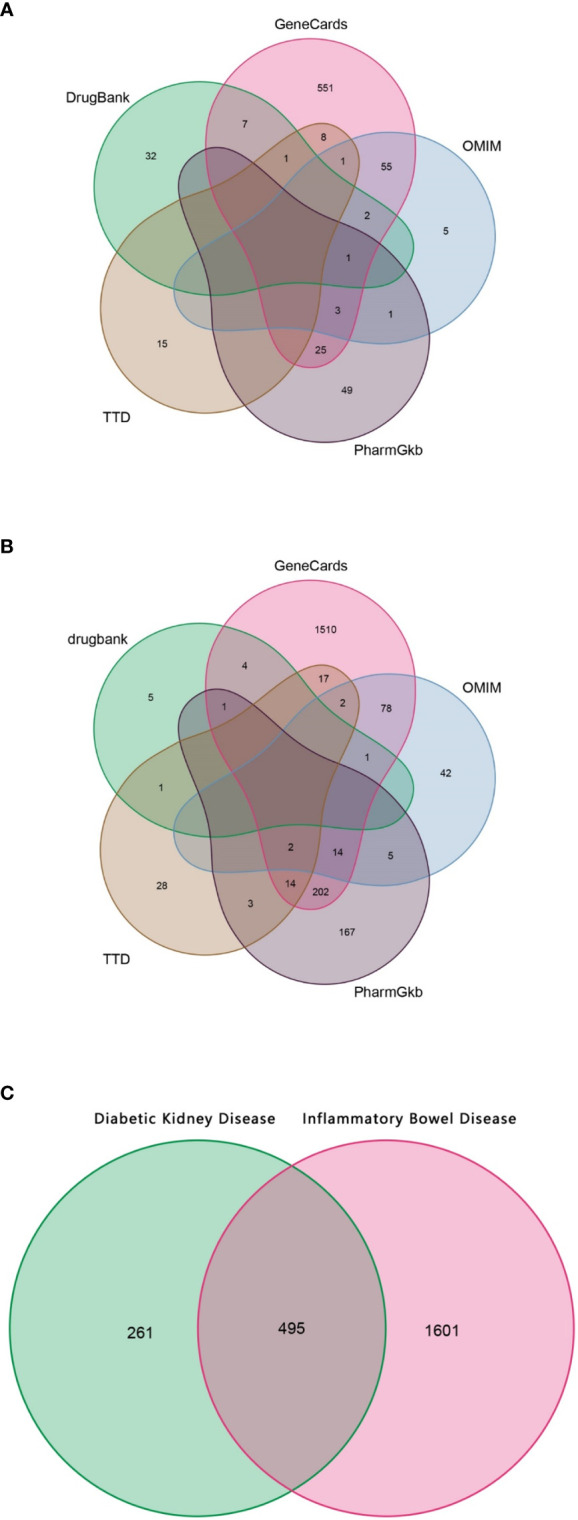
The Venn diagram. **(A)** Collection of gene targets associated with DKD from databases. **(B)** Collection of gene targets associated with IBD from databases. **(C)** Identification of the possible risk genes of DKD complicated with IBD.

Using “diabetic nephropathy” and “inflammatory bowel disease” as keywords, their related genes were retrieved from OMIM, GeneCards, PharmGkb, TTD, and DrugBank databases, respectively. As shown in **Figure 2A**, a total of 756 genes related to DKD were collected, and 2,096 genes related to IBD were retrieved, as shown in **Figure 2B**. For both DKD and IBD, the GeneCards database is the most common source of disease-related genes, suggesting that the it has the advantage of being comprehensive in terms of human genetic information.

### Identification of the risk genes for DKD with IBD

Since the targeted genes for both DKD and IBD were gathered, using the shared genes described above, 459 possible risk genes for DKD complicated with IBD were obtained, as shown in **Figure 2C**. A total of 459 disease-associated risk genes are obviously too many, suggesting that further screening for key risk genes among them is necessary.

### Risk genes for DKD with IBD’ PPI network

**Figure 3:**
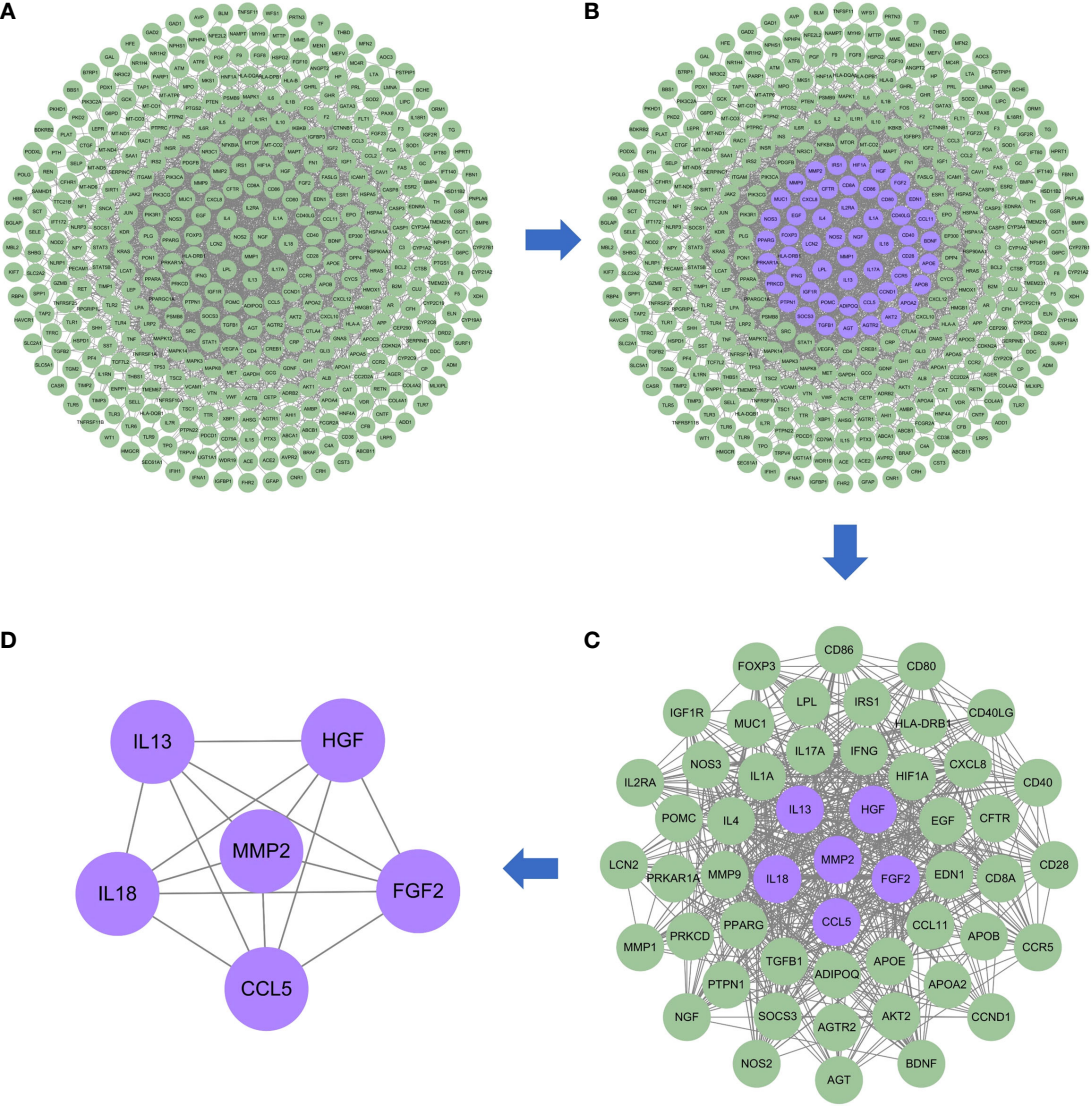
Protein–protein interaction network of the possible risk genes of DKD complicated with IBD. **(A)** The original PPI network of therapeutic targets and all nodes in the network are green. **(B)** The core nodes in this network screened by their topological features are purple, and the other nodes are green. **(C)** The sub-network constructed from the core nodes of B, the core nodes in this network screened by their topological features are purple, and the other nodes are green. **(D)** The sub-network constructed from the core nodes of C, all nodes in the network are purple and represent the final central therapeutic targets obtained through screening.

The PPI network of risk genes for DKD with IBD is shown in **Figure 3A**, including 407 nodes and 2,330 edges. Four topological features of 407 targets were calculated using a network analyzer to identify key nodes in the network. The detailed information of all the risk genes in the PPI network is displayed in **Supplementary Table S1**. The median values of the degree, node betweenness, closeness, and clustering coefficient were 7, 0.00142208, 0.3137558, and 0.333333, respectively. As shown in **Figures 3B, C**, nodes with values of all four topological parameters exceeding the median were selected to construct subnetworks. In the subnetwork, another selection was performed to finally obtain the central targets (**Figure 3D**). Finally, six genes were identified as central risk genes for DKD with IBD, including matrix metalloproteinase 2 (MMP2), hepatocyte growth factor (HGF), fibroblast growth factor 2 (FGF2), interleukin (IL)-18, IL-13, and C–C motif chemokine ligand 5 (CCL5), and their detailed information in the PPI network is displayed in **Table 1**.

**Table 1:** Central risk genes for diabetic kidney disease with inflammatory bowel disease.

Gene symbol	Protein names	Uniprot ID	Betweenness centrality	Closeness centrality	Degree	Clustering coefficient
MMP2	Matrix metalloproteinase 2	P08253	0.006506	0.366096	24	0.391304
HGF	Hepatocyte growth factor	P14210	0.004258	0.374885	24	0.40942
FGF2	Fibroblast growth factor 2	P09038	0.01366	0.360248	26	0.375385
IL18	Interleukin-18	Q14116	0.004962	0.35961	21	0.42381
IL13	Interleukin-13	P35225	0.002312	0.338616	16	0.425
CCL5	C–C motif chemokine 5	P13501	0.001432	0.346712	15	0.657143

### GO and KEGG pathway enrichment

**Figure 4:**
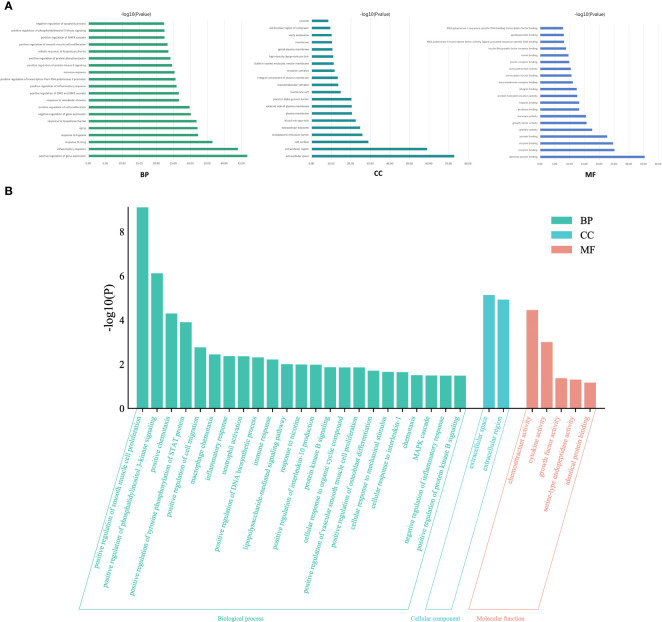
Gene Ontology enrichment analysis. **(A)** The most relevant biological process, cell component, and molecular function terms in all risk genes of DKD complicated with IBD. **(B)** The most relevant biological process, cell component, and molecular function terms in the key risk genes of DKD complicated with IBD.

**Figure 5:**
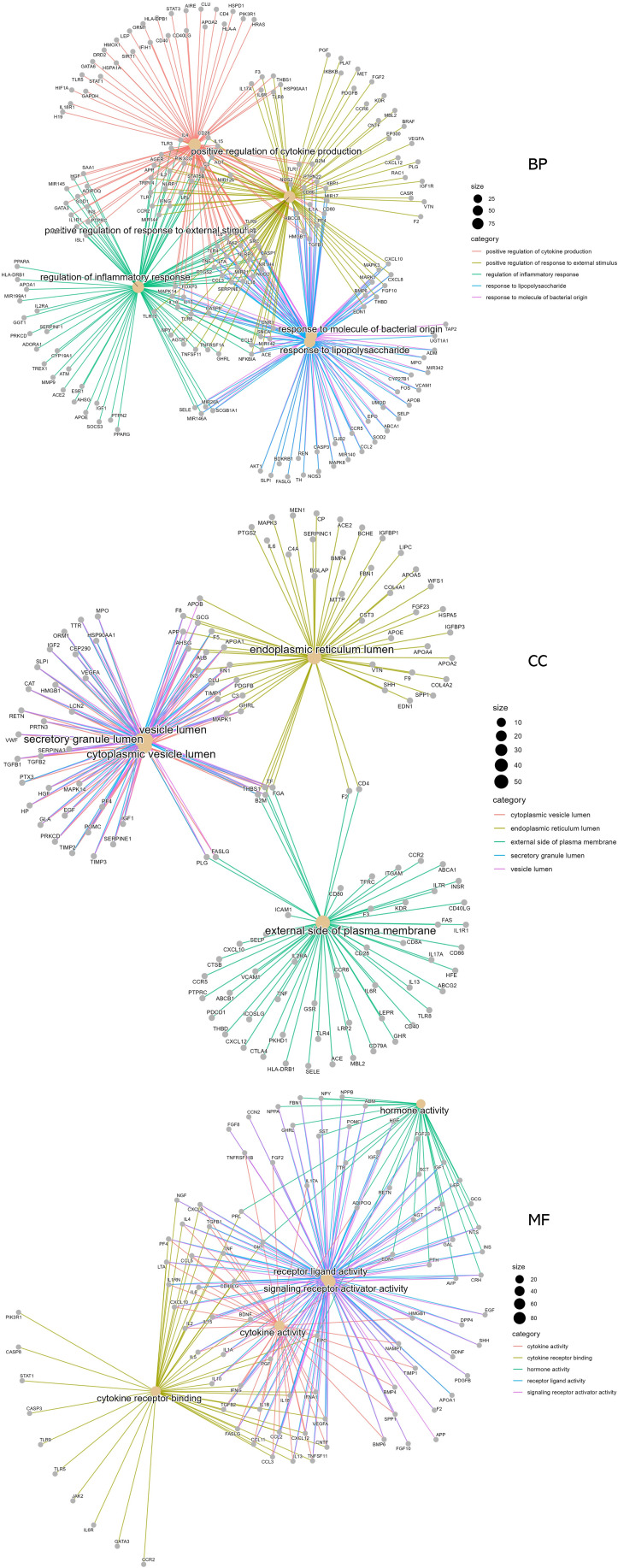
Relationships of the risk genes with the most significant biological process, cell component, and molecular function terms in Gene Ontology enrichment analysis.

To elucidate the complex mechanisms of DKD complicated with IBD, we analyzed the GO biological process (BP), cell component (CC), and molecular function (MF) of 459 possible risk genes and six identified key risk genes, respectively, and the most relevant results that were chosen by *p*-value are presented in **Figures 4A, B**, correspondingly. The specific entries of GO enrichment analysis for BP, CC, and MF of all risk genes and key risk genes are listed in **Supplementary Tables S2, S3**, respectively. The regulation of cytokine production and the regulation of inflammatory response were among the most closely related biological processes, which was in accord with our general cognition to the disease. Moreover, the relationships between the risk genes and the obtained GO enrichment entries are depicted in **Figure 5**.

**Figure 6:**
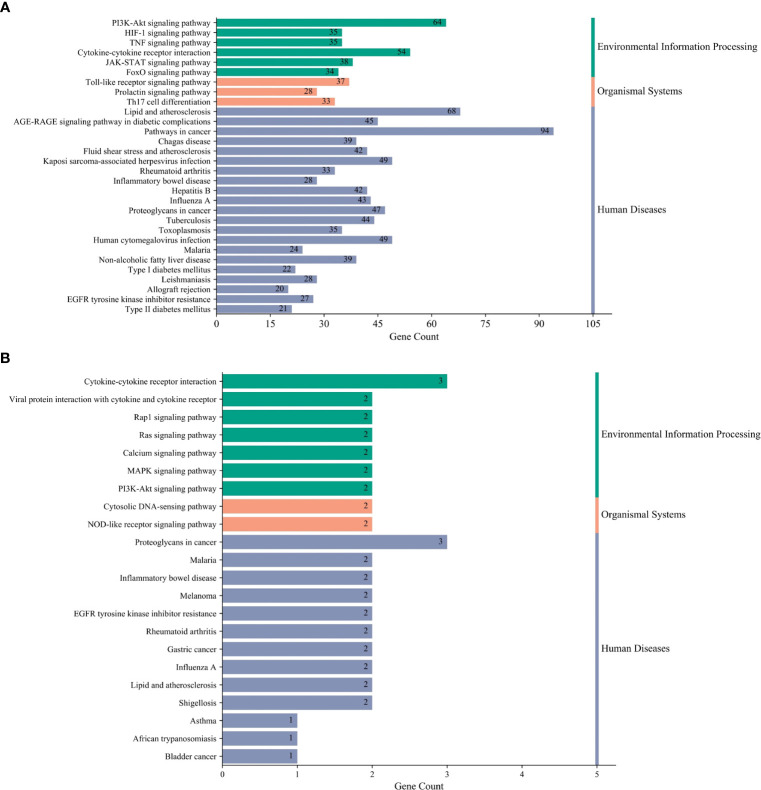
Kyoto Encyclopedia of Genes and Genomes (KEGG) terms. **(A)** The most relevant KEGG terms in all risk genes of DKD complicated with IBD. **(B)** The most relevant KEGG terms in the key risk genes of DKD complicated with IBD.

**Figure 7:**
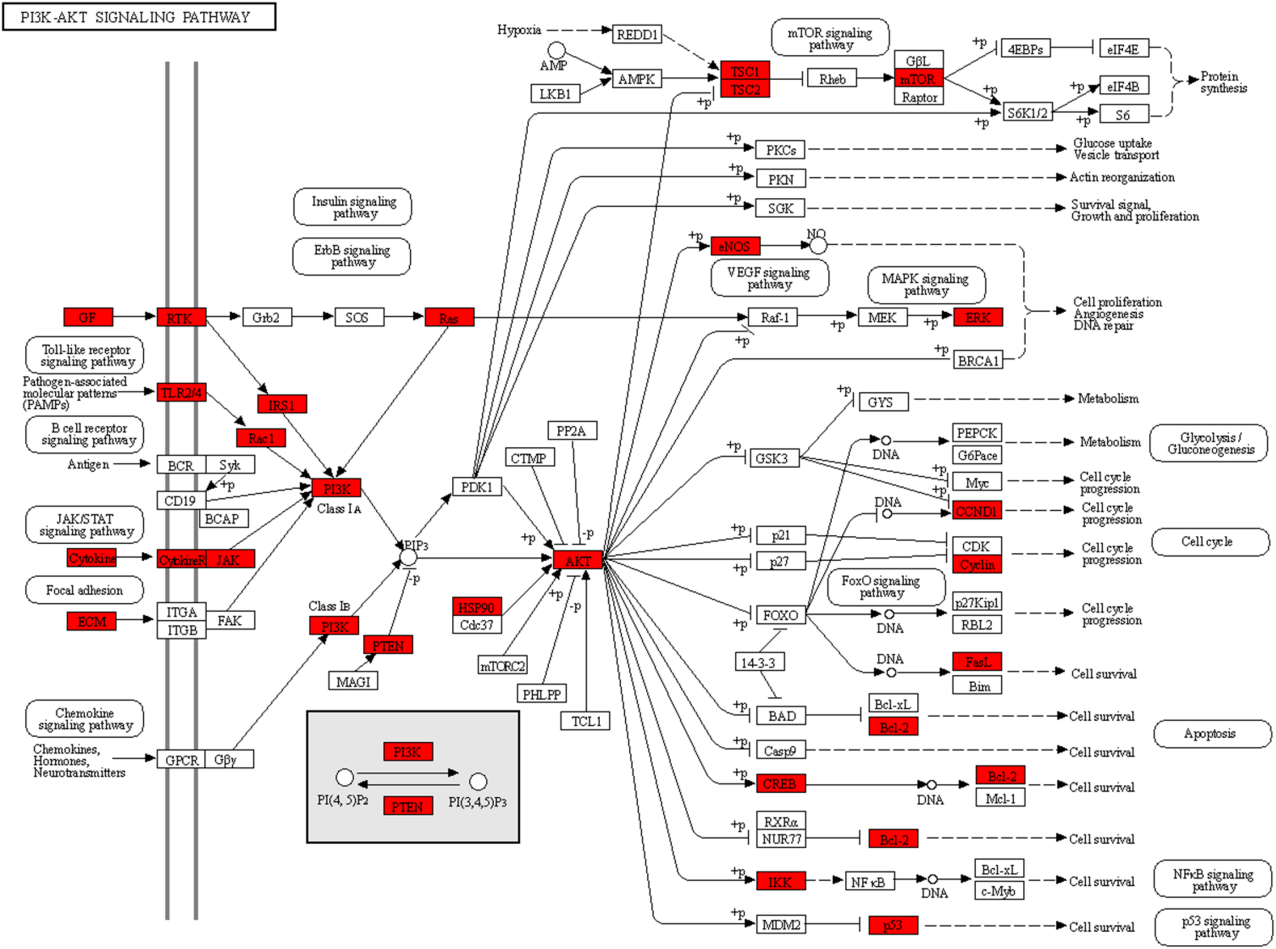
The risk genes for DKD with IBD are depicted in the PI3K-Akt signaling pathway.

To explore the underlying mechanisms of DKD complicated with IBD, KEGG pathway enrichment analysis was also performed on the risk genes and identified key risk genes, respectively, and the most relevant results are presented in **Figures 6A, B**, correspondingly. The detailed information of KEGG enrichment analysis for all risk genes and key risk genes is listed in **Supplementary Tables S4, S5**, respectively. As shown in **Supplementary Table S4** and **Figure 6A**, there were 181 major pathways involved in DKD combined with IBD, *p*<0.05. These 181 pathways were involved in human diseases, pathophysiological mechanisms, and signaling pathways. The top 10 significantly enriched signaling pathways include the advanced glycation end product–receptor for AGE (AGE-RAGE) signaling pathway, Toll-like receptor signaling pathway, PI3K-Akt signaling pathway, hypoxia-inducible factor (HIF)-1 signaling pathway, tumor necrosis factor (TNF) signaling pathway, Janus kinase–signal transducer and activator of transcription (JAK-STAT) signaling pathway, IL-17 signaling pathway, T-cell receptor signaling pathway, mitogen-activated protein kinase (MAPK) signaling pathway, and Rap1 signaling pathway. The risk genes for DKD with IBD in the PI3K-Akt signaling pathway are depicted in **Figure 7**. These results suggest that the pathogenesis of DKD combined with IBD is regulated through the regulation of multiple pathways, and many risk genes for DKD combined with IBD play their roles in multiple pathways simultaneously.

## Discussion

Inflammatory bowel disease (IBD) is an autoimmune disease consisting of Crohn’s disease (CD), ulcerative colitis (UC), and indeterminate colitis. IBD is most common in Western countries, especially in North America and northern Europe; its prevalence is increasing year by year, and its cause is still unclear (20, 21). Genetic, environmental, and host-related factors are associated with the exacerbation of gut inflammation (22). The classical presentations of IBD are malabsorption and gastrointestinal symptoms; however, CD is more likely to manifest as watery diarrhea and vague symptoms, whereas UC is more likely to manifest as diarrhea and bleeding (23). The extraintestinal symptoms and associated diseases of IBD have been increasingly recognized in recent years. Many of these are associated with autoimmune diseases such as type 1 diabetes, thyroid disease, and dermatitis herpetiformis (7). There are many reports of combined kidney disease in patients with IBD, especially DKD (8–10). Therefore, in this study, we applied the bioinformatics approach to investigate the possible mechanisms of IBD complicated with DKD from a genetic perspective.

Through data mining, we found that IBD and DKD have many related genes in common, and MMP2, HGF, FGF2, IL-18, IL-13, and CCL5 were identified as the core risk genes for DKD with IBD by PPI analysis. MMP2 is an enzyme with gelatinase activity. Overexpression of MMP2 in glomerulus and tubules can lead to kidney damage and fibrosis by inflammatory processes through various pathologies (24). Meanwhile, mucosal MMP2 activity has been reported to be upregulated in humans with IBD (25). It facilitates remodeling of ECM and degradation of basal membrane type IV collagen, leading to intestinal ulceration, epithelial damage, and fistula formation (26, 27). HGF is a pleiotropic factor with the activities of antiapoptotic, cytoprotective, and modulating central inflammation and immunoreaction in many diseases (28, 29). Elevated serum HGF level is significantly associated with the incidence of type 2 diabetes (T2DM) and the development of insulin resistance (IR) (30). It has a positive effect on the survival of islet β cells and could protect against high-fat diet-induced obesity and regulate IR (31). Many studies have also found that serum HGF levels are higher in IBD patients compared with controls, which may be a good acute phase response biomarker of IBD activity (32, 33). HGF could modulate intestinal epithelial cell proliferation and migration, thus accelerating the repair of the intestinal mucosa (34). Recombinant human HGF and HGF gene therapy eliminates the severity in some animal models of IBD and is considered as a new treatment modality (35). IL-18 and IL-13 are both inflammatory cytokines, and CCL5 is a chemokine; inflammatory processes have been demonstrated to be associated with the development of DKD (36). It has been reported that IL-18 levels in urine and serum are increased in patients with DKD, and the serum IL-18 level is related to renal injury severity in DKD (37, 38). IL-18 could induce the release of interferon γ; lead to the production of other inflammatory cytokines, such as IL-1 and TNF; and induce endothelial cell apoptosis (39). In fact, IL-18 is thought to be more closely related to the progression of DKD than other diabetic complications (40). CCL5 is a potent chemoattractant for macrophages, monocytes, T cells, and granulocytes. CCL5 is significantly upregulated in renal biopsy samples from patients with T2DM and overt nephropathy, and its expression in renal tubular cells has a direct correlation with the interstitial cellular infiltration and the magnitude of proteinuria (41). The production of IL-18 was regarded as a key etiological factor for patients with IBD (42). It has been shown to disrupt the mucosal barrier, trigger inflammation, and amplify damage to the intestinal epithelium during disease (43). CCL5 also plays a crucial role in IBD; its expression is enhanced in the intestine and is closely implicated in the pathophysiology (44–46). These studies on the core risk genes for DKD with IBD validate our findings to some extent. These core risk genes may serve as the potential therapeutic targets for the development of new drugs and possible diagnostic biomarkers for the clinical identification of DKD complicated with IBD.

According to the KEGG terms, the risk genes for DKD complicated with IBD were mainly associated with the PI3K-Akt signaling pathway, AGE-RAGE signaling pathway in diabetic complications, and HIF-1 signaling pathway. Many studies have shown that stimulation of angiogenesis is an active participant in the establishment and maintenance of tissue inflammation in IBD (47, 48). The study by Zhou et al. found that the PI3K-Akt signaling pathway plays a crucial role in the angiogenesis of IBD, which could be activated by placental growth factor and induce the angiogenic effects in IBD (49). In addition, pro−inflammatory cytokines, such as tumor necrosis factor (TNF), are pivotal for the development of IBD (50). The activation of TNF receptor could stimulate the production of reactive oxygen species, which mediates the cell fate regarding senescence and apoptosis through the PI3K/AKT pathway (51). Decreased phosphorylation of PI3K-Akt could give rise to podocytes apoptosis, and many drugs have been reported to exert anti-apoptosis and renal protection effects in DKD through activating PI3K-Akt signaling pathway (52, 53). Therefore, it is reasonable that the dysregulation of the PI3K/AKT pathway is implicated in the induction and progression of DKD complicated with IBD. Hypoxia-induced activation of the HIF signaling pathway is a common feature of inflammatory diseases, including IBD (54). Hypoxia-inducible factor 1-alpha (HIF-1α) acts as a transcription factor that regulates cellular adaptation to low oxygen levels and supports the development and function of the gut barrier, thereby conferring protection against IBD (55), while constitutive activation of HIF-2α leads to the development or exacerbation of IBD in colitis models (56). In DKD, tubular HIF activity is inhibited, and HIF activation protects mitochondrial function and prevents diabetes-induced tissue hypoxia, tubulointerstitial fibrosis, and proteinuria (57). These studies on the signaling pathway for DKD with IBD also validate our findings to some extent.

Our study found that DKD and IBD share many common related genes and pathogenic mechanisms, oxidative stress, chronic inflammatory response, and immune dysfunction were possible mechanisms, and further research on these common targets for DKD complicated with IBD, especially the core targets, is important for the development of new effective drugs and the early diagnosis. Our study is based on the data mining and bioinformatics approach and has limitations due to the lack of experimental validation, mainly in the failure to validate the expression of the identified key risk genes in the blood of the patients with DKD combined with IBD and the changes in potential signaling pathways. Therefore, experiments on the key targets and related pathways of DKD combined with IBD are also needed to be conducted in the future.

## Data availability statement

The datasets presented in this study can be found in online repositories. The names of the repository/repositories and accession number(s) can be found in the article/**Supplementary Material**.

## Author contributions

XZ performed the research, conducted the bioinformatics analysis, and wrote the first draft of the manuscript. HX, SF, and JY participated in data collection and analysis. YC and YJ participated in the project design and manuscript draft preparation and revision. All the authors have approved the final version of the manuscript.
